# Evaluating the Efficacy of ChatGPT vs. Google Gemini in Generating Patient Education Materials for GLP-1 Receptor Agonists (Semaglutide, Liraglutide, Tirzepatide): A Cross-Sectional Study

**DOI:** 10.7759/cureus.81993

**Published:** 2025-04-10

**Authors:** Nithin Karnan, Sruthi Nair, Farhaan Firoz Fidai, Sri Vidhya Gurrala, Jasmine Salim, Ahmed Gomma

**Affiliations:** 1 Internal Medicine, KAP Viswanathan Government Medical College, Tiruchirappalli, IND; 2 Geriatrics, Birmingham Heartlands Hospital, Birmingham, GBR; 3 Internal Medicine, Katihar Medical College, Katihar, IND; 4 Internal Medicine, NRI Academy of Medical Sciences, Mangalagiri, IND; 5 Internal Medicine, Kims Alshifa Hospital, Perinthalmanna, IND; 6 Internal Medicine, Southeast University, Nanjing, CHN

**Keywords:** artificial intelligence, chatgpt, diabetes, glp-1 receptor agonists, google gemini, liraglutide, patient education, readability analysis, semaglutide, tirzepatide

## Abstract

Introduction: Diabetes management involves using various oral hypoglycemic agents, including new glucagon-like peptide-1 (GLP-1) receptor agonists like semaglutide, tirzepatide, and liraglutide. Artificial intelligence (AI) tools such as ChatGPT (OpenAI, San Francisco, United States) and Google Gemini (Google DeepMind, London, United Kingdom) provide an innovative approach to creating patient education materials, potentially enhancing the accessibility and understanding of medical information. Thus, the study aimed to compare the effectiveness of ChatGPT and Google Gemini in generating patient education brochures for semaglutide, tirzepatide, and liraglutide. Key criteria included readability, similarity, and reliability of the generated content.

Methodology: The cross-sectional study design was conducted in June 2024, involving data collection from ChatGPT-3.5 and Google Gemini. Each AI tool generated educational brochures for the three medications. The responses were evaluated using Flesch-Kincaid readability scores, Quillbot similarity analysis, and a modified DISCERN instrument for reliability assessment. Statistical analysis included univariate t-tests and Pearson’s coefficient of correlation via RStudio v4.3.2 (Posit, Boston, United States).

Results: ChatGPT generated longer brochures with higher word counts compared to Google Gemini, which had better readability scores. Similarity analysis showed that Google Gemini’s content had a higher percentage of overlap. Both AI tools demonstrated high reliability scores, with no significant difference between them.

Conclusions: Google Gemini provided more readable content, while ChatGPT produced slightly more detailed information. Both AI tools were effective in generating reliable patient education materials for GLP-1 receptor agonists. However, future research should incorporate more AI tools and updated versions for comprehensive analysis.

## Introduction

Diabetes is a chronic disease that occurs either when the pancreas does not produce enough insulin or when the body cannot effectively use the insulin it produces [1]. There are various pharmacological drugs available for the treatment of diabetes. Glucagon-like peptide-1 (GLP-1) receptor agonists, or GLP-1 analogs, are one such class of drugs that reduce blood sugar and energy intake by activating the GLP-1 receptor, mimicking the actions of the endogenous incretin hormone GLP-1 that is released after eating by the gut [2]. Semaglutide, tirzepatide, and liraglutide are new oral hypoglycemic drugs classified under this category.

OpenAI, a San Francisco-based company specializing in artificial intelligence (AI) research and development, introduced the generative pre-trained transformer ChatGPT (OpenAI, San Francisco, United States) in November 2022 [3,4]. ChatGPT leverages advanced language processing and machine learning technologies to facilitate conversational interactions with a virtual assistant [4]. On December 6, 2023, Google’s DeepMind launched Gemini (Google DeepMind, London, United Kingdom), which is an AI-model featuring visual language model (VLM) technology and incorporates multiple large language models (LLMs) alongside natural language processing (NLP) capabilities [5,6].

Diabetes, being a chronic metabolic disorder, presents many challenges in optimizing effective care solutions for better patient outcomes [7]. LLMs such as ChatGPT can offer effective strategic solutions to overcome these barriers and attain the best clinical results [8]. AI tools can give us information about GLP-1 agonists in treating diabetes in easy-to-understand language. They can also deliver motivational messages or interventions to encourage adherence to GLP-1 agonist therapy, dietary recommendations, and exercise plans, along with educational resources on how to manage risks. 

ChatGPT and Google Gemini were selected for this study because they are among the most widely used AI language models for generating health-related information. Comparing these two would allow us to assess differences in content quality, readability, and reliability within patient education materials. While other AI tools exist, ChatGPT and Google Gemini were chosen due to their accessibility, popularity, and advanced NLP capabilities.

Aims and objectives

The aims and objectives of the study were to compare ChatGPT and Google Gemini AI tools in generating patient education guides for GLP-1 receptor agonists, such as semaglutide, liraglutide, and tirzepatide, as well as to assess the accuracy and reliability of the patient education materials generated by both AI tools. Additionally, the study sought to evaluate the comprehensiveness and clarity of these materials.

## Materials and methods

Study design and ethical considerations

This original research was a cross-sectional study conducted in June 2024. As the study utilized AI tools alongside the complete absence of human subjects, ethics committee approval was deemed exempt.

Data collection 

For data collection, the main approach was to gather responses from two AI tools: ChatGPT-3.5 and Google Gemini. The purpose was to create patient education brochures introducing three new oral hypoglycemic agents: semaglutide, tirzepatide, and liraglutide. Successively, each of the AI instruments was asked to generate educational tutorials via the following prompts: 

(i) "Write a patient education guide for semaglutide."

(ii) "Write a patient education guide for tirzepatide."

(iii) "Write a patient education guide for liraglutide."

The responses were generated in a single attempt and then collected in a Microsoft Word document (Microsoft Corp., Redmond, United States) for further analysis. 

Data evaluation 

Once the responses were obtained from both AI tools, the content and its accuracy were compared. Various criteria were used for evaluating the responses, including the Flesch-Kincaid calculator, which assessed the word choice, sentence complexity, ease of comprehension, and overall sentence structure to determine the educational material’s readability [9]. 

Similarity analysis was conducted using the Quillbot plagiarism tool (Quillbot Inc., Chicago, United States), which verifies the uniqueness of the newly produced content generated by using AI [10]. 

The reliability of the responses was assessed using the modified DISCERN instrument, adapted to match the assessment of medical information, the credibility of the source, and the reliability of health-related information contained in the brochures [11]. The modified DISCERN score consists of five questions and employs a Likert scale (1-5 points) for each criterion, where higher scores indicate better quality. The total score represents the overall quality and reliability of the evaluated material [11].

Statistical analysis 

In the data analysis, Microsoft Excel (Microsoft Corp., Redmond, United States) and RStudio v4.3.2 (Posit, Boston, United States) were used. For this study, a univariate t-test was applied to test the differences between the responses generated by ChatGPT and Google Gemini, with a predetermined level of significance of p<0.05. 

## Results

ChatGPT and Google Gemini were used to generate brochures for patient education on the medications semaglutide, tirzepatide, and liraglutide.

Table 1 shows the characteristics of the responses generated by ChatGPT and Google Gemini. There was a significant difference in the word count generated by the two AI tools. ChatGPT responses had a significantly higher mean word count (548.70) compared to Google Gemini (437.00), with a p-value of 0.0127. There was no significant difference in the sentence count (p=0.2153) or average word count (p=0.9035) between the two AI tools.

**Table 1:** Characteristics of responses generated by ChatGPT and Google Gemini. *t-test was used to compare the means between ChatGPT and Google Gemini. As per the p-values obtained, there was a statistically significant difference between the words generated by the two AI tools.

Variables	ChatGPT	Google Gemini	p-value (t-test)*
	Mean ± SD	Mean ± SD	
Words	548.70 ± 26.95	437.00 ± 34.18	0.0127*
Sentences	61.00 ± 14.42	46.33 ± 3.79	0.2153
Average words per sentence	9.33 ± 2.18	9.53 ± 1.53	0.9035
Average syllables per word	1.90 ± 0.00	1.77 ± 0.06	0.0572
Grade level	10.47 ± 0.86	9.93 ± 1.10	0.5470
Ease score	36.63 ± 2.24	47.53 ± 5.13	0.0500
Similarity %	26.97 ± 9.28	44.23 ± 18.53	0.2463
Reliability score	4.00 ± 0.00	3.67 ± 0.58	0.4226

Regarding the ease scores, Google Gemini's responses had a higher mean ease score (47.53) compared to ChatGPT (36.63), with a p-value of 0.0500, which is on the threshold of significance. Both AI tools showed similar reliability scores with a p-value of 0.4226, indicating that this difference was not significant.

**Figure 1:**
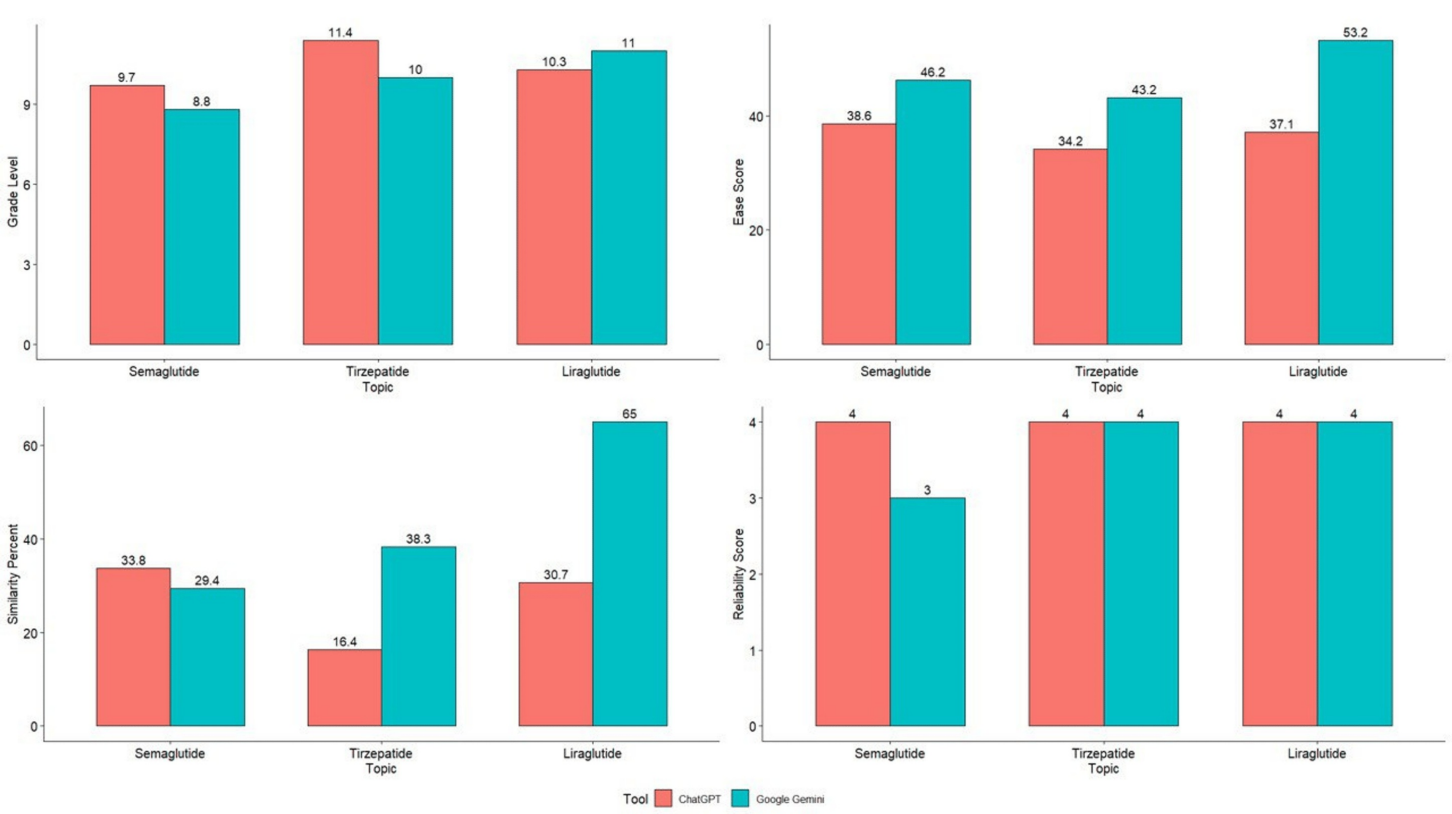
Graphical representation of comparison between grade level, ease score, similarity percentage, and reliability score for the patient education guides generated by ChatGPT and Google Gemini.

Figure 1 presents a graphical representation of the comparison between grade level, ease score, similarity percentage, and reliability score for the patient education guides generated by ChatGPT and Google Gemini.

The grade levels for semaglutide information indicated that ChatGPT generated responses at a grade level of 9.7 compared to Google Gemini's 8.8. For tirzepatide information, ChatGPT had a grade level of 11.4, while Google Gemini's was 10. For liraglutide information, Google Gemini generated responses at a higher grade level (11) compared to ChatGPT (10.3).

In terms of ease scores, Google Gemini's responses about semaglutide were easier to read (46.2) compared to ChatGPT (38.6). For tirzepatide information, Google Gemini had a higher ease score (43.2) compared to ChatGPT (34.2). Similarly, for liraglutide information, Google Gemini’s responses had the highest ease score (53.2) compared to ChatGPT (37.1).

ChatGPT and Google Gemini had similar percentages for similarity (33.8% and 29.4%, respectively), while Google Gemini's responses for tirzepatide had a higher similarity percentage (38.3%) compared to ChatGPT (16.4%). For liraglutide responses, Google Gemini had a significantly higher similarity percentage (65%) compared to ChatGPT (30.7%).

Both AI tools had high reliability scores for semaglutide information, with ChatGPT scoring 4 and Google Gemini scoring 3. Both tools received perfect reliability scores of 4 for tirzepatide and liraglutide information.

## Discussion

A cross-sectional study was conducted to analyze the responses to the patient education brochures on semaglutide, tirzepatide, and liraglutide generated by ChatGPT and Google Gemini.

AI has transformed the healthcare system by improving patient care and positively impacting quality of life. AI encompasses several techniques used to generate information, such as deep learning (DL), NLP, and machine learning (ML). LLMs are among the most commonly used techniques for generating extensive amounts of information [12]. Both physicians and patients access AI, which produces information that is easy-to-understand without delving too deeply into the details [13]. It serves as a very informative tool for producing individualized data that aids in patient decision-making and provides personalized assistance [14].

In this study, there was no significant difference in the ease score (p=0.05) for the brochures generated by both AI tools; however, the ease score was better for Google Gemini. A similar comparative study analyzed the readability and quality of data generated by Google Gemini and ChatGPT-4 using the Flesch-Kincaid scale. It concluded that ChatGPT-4 had a higher grade level (p=0.003) and a lower ease score (p=0.005). Thus, ChatGPT-4 produced accurate data suited for individuals with higher levels of education [15]. In another retrospective cross-sectional study, the ease score and quality of data generated by ChatGPT for general questions regarding common retinal conditions were assessed. It concluded that while the data generated were accurate, they were relatively difficult for an average high school patient to understand. This is because the patient education literature accessed by AI tools is often complex and requires a higher level of understanding [16].

This study observed a significant difference in the word count generated by both AI tools. The practical implications of these differences highlight the need to balance comprehensiveness with clarity when using AI-generated medical information. Future research should explore whether a higher word count correlates with improved patient understanding or if more targeted, succinct content is preferable.

In the field of research, there has been an increase in plagiarism; a review assessing 14,719 articles concluded that there was a high percentage of plagiarism (44.9%). This was attributed to the vast amounts of data available on Google and the lack of investigations into cases of plagiarism [17]. In this study, no significant difference was found between the AI tools for similarity percentage (p=0.2463). However, Google Gemini had a higher percentage of plagiarism. This finding contrasts with a systematic review of 60 articles, which reported that ChatGPT exhibited a very high level of plagiarism (96.7%) in the information it generated [18].

The modified DISCERN score is used to grade the reliability of the information generated by online articles. While the average DISCERN score in this study was not statistically significant, it was higher for ChatGPT compared to Google Gemini. This can be attributed to ChatGPT being trained on vast amounts of data from articles, books, and Wikipedia, whereas Google Gemini relies on information from web searches only. This finding can be compared to an observational study that assessed the reliability of three major AI tools (Google's AI Bard, ChatGPT-3.5, and Bing AI), which found that Bard (46.3 ± 2.8) had a significantly higher DISCERN score compared to ChatGPT-3.5 and Bing AI [19].

Limitations

The methodology employed in data collection involved generating responses from both ChatGPT and Google Gemini only once for each prompt. While this provided a comparative snapshot of the AI tools, it may not fully account for variability in the generated responses. Repeating the data collection multiple times could offer a more robust representation of each tool's performance and help reduce any potential biases or inconsistencies. Additionally, the study focused only on two AI tools; future research should consider evaluating a broader range of AI models for comparison. Furthermore, the information generated by these tools should be verified and updated regularly to ensure alignment with the latest medical guidelines, enhancing the accuracy and relevance of the content produced for patient education. 

## Conclusions

To conclude, there was a significant difference in the word count generated by both AI tools for the patient education brochures on semaglutide, tirzepatide, and liraglutide. There was no correlation in the ease score, grade level, and reliability score between the two AI tools. Future research should broaden its scope by incorporating more AI tools and newer medications for diabetes mellitus and other endocrine disorders. Additionally, it is essential to ensure that AI-generated information aligns with the latest clinical guidelines to enhance accessibility and reliability for both patients and healthcare providers.

## References

[1] Bastaki S (2005). Diabetes mellitus and its treatment. Int J Diabetes Metab.

[2] Verspohl EJ (2009). Novel therapeutics for type 2 diabetes: incretin hormone mimetics (glucagon-like peptide-1 receptor agonists) and dipeptidyl peptidase-4 inhibitors. Pharmacol Ther.

[3] Shneiderman B (2020). Bridging the gap between ethics and practice: guidelines for reliable, safe, and trustworthy human-centered AI systems. ACM Trans Interact Intell Syst.

[4] King MR (2023). A conversation on artificial intelligence, chatbots, and plagiarism in higher education. Cell Mol Bioeng.

[5] Imran M, Almusharraf N (2024). Google Gemini as a next generation AI educational tool: a review of emerging educational technology. Smart Learn Environ.

[6] Farrokhnia M, Banihashem SK, Noroozi O, Wals A (2024). A SWOT analysis of ChatGPT: implications for educational practice and research. Innov Educ Teach Int.

[7] Iyengar V, Wolf A, Brown A, Close K (2016). Challenges in diabetes care: can digital health help address them?. Clin Diabetes.

[8] Hacking S (2024). ChatGPT and medicine: together we embrace the AI renaissance. JMIR Bioinform Biotechnol.

[9] Flesch R (2017). Flesch-Kincaid readability test. https://rockstar-english.com/lessons/advanced/12-Flesch_Kincaid_Readability_Test.pdf.

[10] Fitria TN (2021). QuillBot as an online tool: students’ alternative in paraphrasing and rewriting of English writing. Englisia.

[11] Li HO, Bailey A, Huynh D, Chan J (2020). YouTube as a source of information on COVID-19: a pandemic of misinformation?. BMJ Glob Health.

[12] Alowais SA, Alghamdi SS, Alsuhebany N (2023). Revolutionizing healthcare: the role of artificial intelligence in clinical practice. BMC Med Educ.

[13] Arfat Y, Mittone G, Esposito R, Cantalupo B, DE Ferrari GM, Aldinucci M (2022). Machine learning for cardiology. Minerva Cardiol Angiol.

[14] Robinson CL, D’Souza RS, Yazdi C, Diejomaoh EM, Schatman ME, Emerick T, Orhurhu V (2024). Reviewing the potential role of artificial intelligence in delivering personalized and interactive pain medicine education for chronic pain patients. J Pain Res.

[15] Mastrokostas PG, Mastrokostas LE, Emara AK (2024). GPT-4 as a source of patient information for anterior cervical discectomy and fusion: a comparative analysis against Google web search. Global Spine J.

[16] Momenaei B, Wakabayashi T, Shahlaee A (2023). Appropriateness and readability of ChatGPT-4-generated responses for surgical treatment of retinal diseases. Ophthalmol Retina.

[17] Armond AC, Gordijn B, Lewis J, Hosseini M, Bodnár JK, Holm S, Kakuk P (2021). A scoping review of the literature featuring research ethics and research integrity cases. BMC Med Ethics.

[18] Sallam M (2023). ChatGPT utility in healthcare education, research, and practice: systematic review on the promising perspectives and valid concerns. Healthcare (Basel).

[19] Seth I, Lim B, Xie Y, Cevik J, Rozen WM, Ross RJ, Lee M (2023). Comparing the efficacy of large language models ChatGPT, BARD, and Bing AI in providing information on rhinoplasty: an observational study. Aesthet Surg J Open Forum.

